# Disseminated Histoplasmosis in a Patient with Myelofibrosis on Ruxolitinib: A Case Report and Review of the Literature on Ruxolitinib-Associated Invasive Fungal Infections

**DOI:** 10.3390/jof10040264

**Published:** 2024-03-31

**Authors:** Chia-Yu Chiu, Teny M. John, Takahiro Matsuo, Sebastian Wurster, Rachel S. Hicklen, Raihaan Riaz Khattak, Ella J. Ariza-Heredia, Prithviraj Bose, Dimitrios P. Kontoyiannis

**Affiliations:** 1 Department of Infectious Diseases, Infection Control, and Employee Health, The University of Texas MD Anderson Cancer Center, Houston, TX 77030, USA; chiu.chia-yu@mayo.edu (C.-Y.C.); tmjohn1@mdanderson.org (T.M.J.); tmatsuo@mdanderson.org (T.M.); stwurster@mdanderson.org (S.W.); raihaankhattakmd@gmail.com (R.R.K.); eariza@mdanderson.org (E.J.A.-H.); 2 Research Medical Library, The University of Texas MD Anderson Cancer Center, Houston, TX 77030, USA; rshicklen@mdanderson.org; 3 Department of Leukemia, Division of Cancer Medicine, The University of Texas MD Anderson Cancer Center, Houston, TX 77030, USA; pbose@mdanderson.org

**Keywords:** ruxolitinib, fedratinib, Janus kinase inhibitor, myelofibrosis, invasive fungal infection, histoplasmosis

## Abstract

Ruxolitinib, a selective inhibitor of Janus kinases, is a standard treatment for intermediate/high-risk myelofibrosis (MF) but is associated with a predisposition to opportunistic infections, especially herpes zoster. However, the incidence and characteristics of invasive fungal infections (IFIs) in these patients remain uncertain. In this report, we present the case of a 59-year-old woman with MF who developed disseminated histoplasmosis after seven months of ruxolitinib use. The patient clinically improved after ten weeks of combined amphotericin B and azole therapy, and ruxolitinib was discontinued. Later, the patient received fedratinib, a relatively JAK2-selective inhibitor, without relapse of histoplasmosis. We also reviewed the literature on published cases of proven IFIs in patients with MF who received ruxolitinib. Including ours, we identified 28 such cases, most commonly due to *Cryptococcus* species (46%). IFIs were most commonly disseminated (39%), followed by localized lung (21%) infections. Although uncommon, a high index of suspicion for opportunistic IFIs is needed in patients receiving JAK inhibitors. Furthermore, the paucity of data regarding the optimal management of IFIs in patients treated with JAK inhibitors underscore the need for well-designed studies to evaluate the epidemiology, pathobiology, early diagnosis, and multimodal therapy of IFIs in patients with hematological malignancies receiving targeted therapies.

## 1. Introduction

Myelofibrosis (MF) is the most advanced chronic Philadelphia chromosome-negative myeloproliferative neoplasm (MPN). It may present as primary myelofibrosis (PMF) or evolve from essential thrombocythemia (ET; post-ET MF) or polycythemia vera (PV; post-PV MF) [1]. Ruxolitinib, an orally bioavailable potent and selective inhibitor of Janus kinases (JAK) 1 and 2, has been approved for the treatment of MF and PV [2]. Ruxolitinib has been shown to reduce spleen size, decrease symptoms, and improve survival in patients with MF [3].

However, ruxolitinib exerts potent anti-inflammatory and immunosuppressive effects and has been associated with infectious complications, most commonly urinary tract infection (4.7–24.6%), pneumonia (5.3–13.1%), herpes zoster (1.9–11.5%), and sepsis (1.3–7.9%) [4]. Other opportunistic infections reported in these patients include tuberculosis, toxoplasmosis, progressive multifocal leukoencephalopathy, and hepatitis B reactivation [5,6]. Although opportunistic fungal infections (e.g., cryptococcosis and *Pneumocystis jirovecii* pneumonia) have been reported in MF patients receiving ruxolitinib [5], the incidence and characteristics of invasive fungal infections (IFIs) in these patients remain uncertain.

Herein, we present a patient with MF who developed disseminated histoplasmosis after seven months of ruxolitinib treatment. Additionally, we critically review the literature on all published cases of proven IFIs in MF patients who received ruxolitinib.

## 2. Case Summary

A 59-year-old woman presented to our hospital in April 2023 with one month of fever. She is originally from Pakistan and immigrated to the USA 30 years ago. She had a history of PV, was diagnosed in March 2019, and was treated with acetylsalicylic acid and hydroxyurea. In August 2022, she was diagnosed with MF on bone marrow biopsy after presenting with progressive weight loss, fatigue, early satiety, and splenomegaly (19 cm in craniocaudal dimension). Ruxolitinib was started at 10 mg twice daily and was increased to 15 mg twice daily one month later. In January 2023, the patient experienced an episode of *Legionella* pneumonia that was treated with levofloxacin on an outpatient basis. At the beginning of February 2023, she went to Puerto Vallarta, Mexico, for a 3-day trip and reported seeing a few bats at the hotel restaurant. At the end of February 2023, she developed persistent high-grade fever (104 °F) associated with fatigue, dry cough, weight loss, and poor appetite. She was admitted to an outside hospital and received empiric antibiotic therapy with cefepime plus doxycycline. An extensive infectious disease workup was negative, including human immunodeficiency virus, syphilis, hepatitis A, B, and C, *Bartonella* serology, *Brucella* serology, Q fever serology, typhus serology, galactomannan, (1,3)-beta-D-glucan, *Cryptococcus* serum antigen, cytomegalovirus plasma quantitative polymerase chain reaction (PCR), Epstein–Barr virus plasma quantitative PCR, and blood cultures. Bone marrow biopsy revealed MF with negative Gram, Giemsa, and acid-fast stains. The patient’s fever slightly improved during her hospital stay, and she was discharged with prednisone (20 mg twice daily) for anti-inflammatory purposes as her infectious disease workup was negative. 

**Figure 1:**
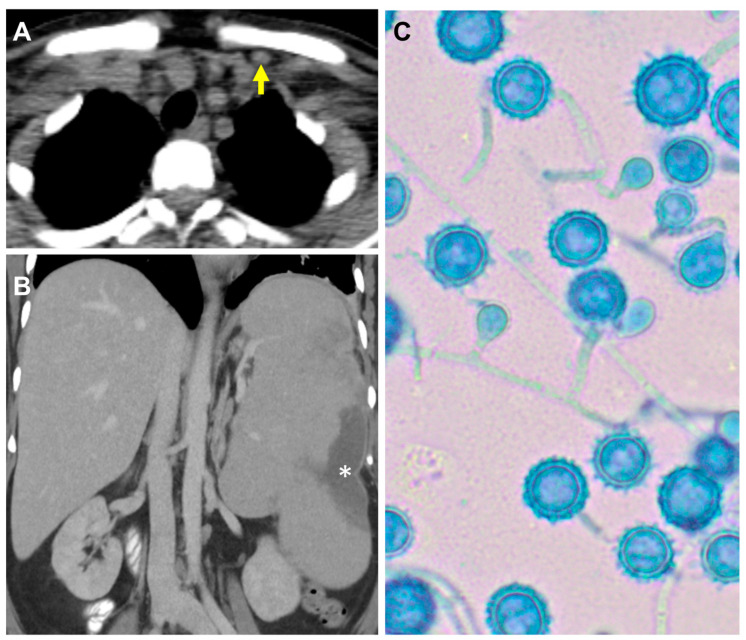
A 59-year-old woman with myelofibrosis and disseminated histoplasmosis after 7 months of ruxolitinib. (**A**) Left subclavicular lymphadenopathy (yellow arrow). (**B**) Splenomegaly (spleen, 19 cm in craniocaudal dimension) with splenic infarction (white asterisk). (**C**) *Histoplasma* recovered from bone marrow culture (×400 magnification).

A few days later, the patient was readmitted to our hospital (MD Anderson Cancer Center, Houston, TX, USA) with severe epigastric pain and recurrent non-neutropenic fever despite prednisone. Computed tomography (CT) showed cervical lymphadenopathy, the largest lymph node being in the left subclavicular region (Figure 1A), and hepatosplenomegaly with splenic infarction (Figure 1B). At that time, an outside hospital report of a positive *Histoplasma* urine antigen (>20 ng/mL; MiraVista diagnostics, Indianapolis, IN, USA) and recovery of *Histoplasma capsulatum* from bone marrow culture was received. Repeat infectious disease workup that included *Aspergillus* antigen and *Cryptococcus* serum antigen was negative. The (1,3)-beta-D-glucan level was borderline positive at 80 pg/mL. Her urine *Histoplasma* antigen (Mayo Clinic Laboratories, Rochester, MN, USA) was negative. A repeat bone marrow biopsy, fungal blood culture, and cervical lymph node biopsy were performed, and all grew *Histoplasma capsulatum* (Figure 1C). This was identified as a member of Latin American group A by whole-genome sequencing at the Fungus Testing Laboratory, University of Texas Health Science Center in San Antonio. 

**Figure 2:**
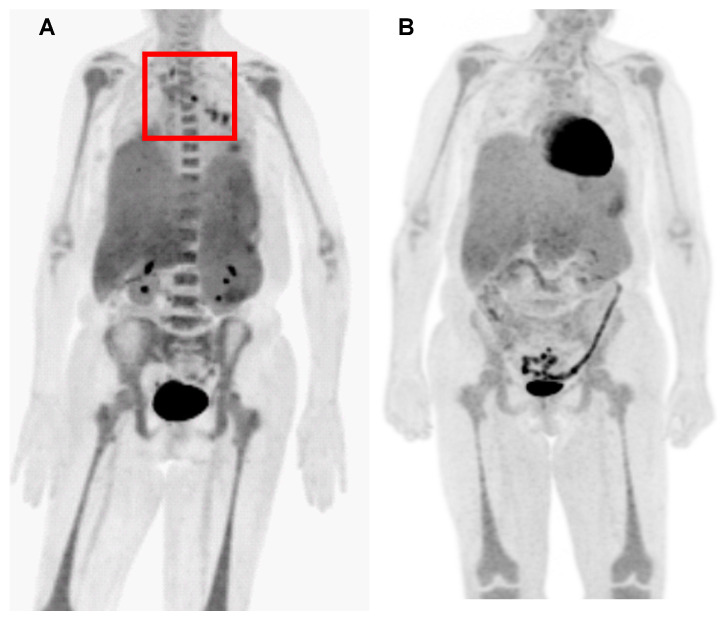
Serial PET scan in our patient with disseminated histoplasmosis. (**A**) PET showed newly developed mediastinum lymphadenopathy (red square) while the patient was receiving intravenous liposomal amphotericin B (3 mg/kg/daily) and oral itraconazole. (**B**) After switching to posaconazole and increased liposomal amphotericin B (5 mg/kg/daily), follow-up PET showed decreasing F-18 FDG avidity of the mediastinal and hilar lymph nodes. Abbreviation: PET—positron emission tomography.

Once the patient was diagnosed with disseminated histoplasmosis, intravenous (IV) liposomal amphotericin B (3 mg/kg/day) was started immediately, and itraconazole (200 mg oral capsule, twice daily) was added 5 days later. After initial improvement of the fever, the patient was discharged on this antifungal regimen. She was readmitted shortly thereafter with a recurrent fever of 102 °F and persistent abdominal pain. Positron emission tomography (PET) revealed newly developed mediastinal lymphadenopathy (Figure 2A) and stable hepatosplenomegaly. Liposomal amphotericin B was increased to 5 mg/kg/day, and oral itraconazole was switched to oral posaconazole (300 mg tablet daily). Her fever subsequently subsided, and she was discharged.

The patient subsequently developed generalized edema, acute kidney injury, and new onset hypertension while on liposomal amphotericin B and posaconazole. Therefore, the IV liposomal amphotericin B dose was decreased to 5 mg/kg three times per week. Her serum posaconazole and potassium levels were 1200 ng/mL and 3.7 mEq/L, respectively. Liposomal amphotericin B was discontinued and posaconazole was switched to isavuconazole due to concern of posaconazole-induced pseudoaldosteronism. Hypertension, weight gain, and hypokalemia abated after the switch from posaconazole to isavuconazole. Ruxolitinib was discontinued following the diagnosis of histoplasmosis in June 2023. In September 2023, the patient was started on fedratinib, a relatively JAK2-selective inhibitor approved for the treatment of MF, which was tolerated without any significant adverse events or relapse of histoplasmosis to date.

**Figure 3:**
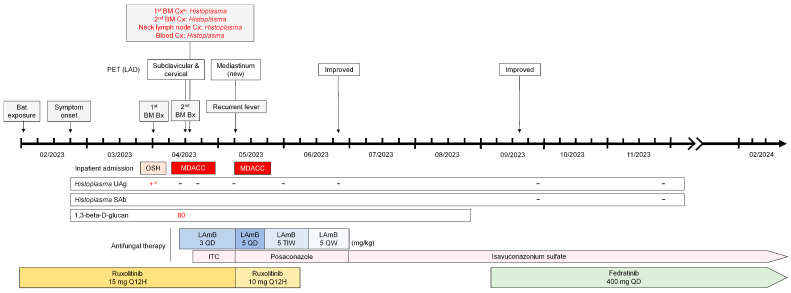
The clinical course of the presented case. ^a.^ MDACC providers were made aware of this result. Abbreviations: +—positive; −—negative; BM Bx—bone marrow biopsy; Cx—culture; ITC—itraconazole; LAD—lymphadenopathy; LAmB 3 QD—liposomal amphotericin B 3 mg/kg/day; LAmB 5 QD—liposomal amphotericin B 5 mg/kg/day; LAmB 5 TIW—liposomal amphotericin B 5 mg/kg three times per week; LAmB 5 QW—liposomal amphotericin B 5 mg/kg weekly; MDACC—MD Anderson Cancer Center; OSH—outside hospital; PET—positron emission tomography; SAb—serum antibody; Q12H—every 12 h; QD—daily; UAg—urine antigen.

On her 10th month of antifungal therapy, the patient maintained a complete clinical and radiologic response (i.e., serial follow-up PETs showed decreased F-18 FDG avidity of lymph nodes) (Figure 2B). Multiple serum Histoplasma serologies (complement fixation and immunodiffusion assay) and urine Histoplasma antigen tests (Mayo Clinic Laboratories, MN, USA) have been negative since April 2023 (Figure 3). As of February 2024, the patient continues to receive antifungal monotherapy with isavuconazole (Figure 3). 

## 3. Case Discussion and Systematic Review of the Literature

A comprehensive literature review was conducted to identify patients with MF who developed IFIs after receiving ruxolitinib. From inception to February 2024, OVID-Embase, OVID-MEDLINE, PubMed, and Scopus were queried using both natural language and controlled vocabulary terms for myelofibrosis, ruxolitinib, and fungal infections. The full search strategy is available in Supplementary Materials. We also searched the reference lists of all relevant publications for additional references. We included cases that provided adequate information regarding the diagnosis, treatment, and outcome of IFIs. 

The reported incidence of IFIs in patients who received ruxolitinib for MF was 0.6–2.6% but increased to 3.7–4.9% in mixed cohorts of patients with ET, PV, MF, and stem cell transplant recipients who receive ruxolitinib as a treatment for graft-vs-host disease (GVHD) (Table 1).

The literature review identified 28 MF patients (including ours) who had proven IFIs while receiving ruxolitinib [5,10,12,13,14,15,16,17,18,19,20,21,22,23,24,25,26,27,28,29,30,31,32]. The patients’ median age was 70 years (interquartile range [IQR], 65–73). Most patients were male (69%) and had primary MF (54%). The median time from ruxolitinib initiation to IFI onset was 11 months (IQR 6–27). IFIs were most commonly disseminated (39%), followed by localized lung (21%) and central nervous system (18%) infections. The most common pathogens were *Cryptococcus* spp. (46%), *Coccidioides* spp. (11%), and *Pneumocystis jirovecii* (11%). In most cases, ruxolitinib was discontinued permanently (76%) or put on hold (18%). No patients had received antifungal prophylaxis at IFI diagnosis. Five patients (18%) had co-occurring infections, including one case each of *Klebsiella pneumoniae* bacteremia, pulmonary tuberculosis, pneumonia due to *Mycobacterium avium* complex, and *Mycobacterium haemophilum* skin infection. One patient had disseminated tuberculosis and shingles (herpes zoster) (Table 2). 

The mechanism of action of ruxolitinib may explain the occurrence of IFIs. Ruxolitinib inhibits JAK-1 and JAK-2 acts on downstream Signal Transducer and Activators of the Transcription (STAT) pathways. JAK-STAT pathways are crucial for the activation and differentiation of various innate (e.g., dendritic cells, natural killer cells) and adaptive (e.g., type-1 and type-17 T-helper cells) immune cell subsets that are pivotal effectors and orchestrators of antifungal host defense [4,33]. Specifically, animal studies have shown that the interferon-gamma–JAK1/2–STAT1 axis plays an essential role in the protective response against cryptococcosis via STAT1-mediated activation of classical (M1) macrophages and effective type-1 T-helper cell responses [34,35]. This might explain why *Cryptococcus* infection is the dominant IFI in patients who receive ruxolitinib. Furthermore, JAK1/2 inhibitors lead to decreased production of cytokines, including interleukins (e.g., IL-6), interferon-gamma, tumor necrosis factor-alpha, and granulocyte-macrophage colony-stimulating factor, and also affect downstream receptor signaling in response to cytokine stimuli (e.g., IL-2) [34,35]. These cytokines are well-known “master regulators” of antifungal defense against all classes of pathogenic fungi and have pleiotropic roles in immune cell recruitment, differentiation, and maturation, as well as the initiation of antifungal effector responses by neutrophils and mononuclear phagocytes [33]. Despite these relatively well-understood molecular mechanisms, determinants of individual IFI risk and the relevance of other factors (such as age, comorbidities, pre-existing cytopenia, previous treatments, concurrent immunosuppressive therapy, and intensity of exposure to fungi) and possible immunogenetic defects remain unclear. 

The overall infection rate was not significantly different between ruxolitinib and the control groups (placebo or best available therapy) in phase 3 clinical trials of patients with various MPNs, except for a significantly higher risk of herpes zoster infection in patients receiving ruxolitinib [5]. This observation was made in both MF patients (COMFORT I and COMFORT II trials) [9,36] and PV patients (RESPONSE and RESPONSE-2 trials) [37,38]. In contrast, the largest national registry, which included 837 patients with MF in Sweden, showed an increased risk of infection with ruxolitinib compared to other cytoreductive therapies [39]. It remains controversial whether the observed increased risk of infection in MF is more inherent to the underlying hematologic disorder or related to ruxolitinib. Interestingly, we found only two published cases of PV (not included in Table 2) that developed IFIs when receiving ruxolitinib [40,41] but 28 MF patients who developed proven IFIs when receiving ruxolitinib (Table 2). However, given the limited number of IFIs reported in patients who received ruxolitinib, it remains unclear whether the underlying hematologic disease (PV vs. MF) influences the risk of IFIs.

One retrospective study showed that 123 out of 446 MF patients experienced infectious events after a median ruxolitinib exposure of 24 months [8]. In patients with MF on ruxolitinib, both a prior history of infectious events and a high international prognostic scoring system (IPSS) score were significantly correlated with higher infectious risk [8]. Interestingly, the infection rate decreased over time with ruxolitinib exposure, and the total cumulative dose of ruxolitinib was not correlated with infection risk [8]. Furthermore, spleen reduction ≥ 50% from baseline after 3 months of ruxolitinib treatment was associated with better infection-free survival [8]. These findings reinforce the concept that MF disease severity is an important risk factor for infections, and the therapeutic effect of ruxolitinib in reducing splenomegaly might reduce infection risk. Notably, 39% of the cases in our review had documented splenomegaly at the onset of IFIs, which might imply that poor oncologic response to ruxolitinib could be a critical modulator of IFI risk in these patients.

It is important to recognize that IFIs can occur at any time after ruxolitinib treatment is initiated, as indicated by the broad IQR of 6–27 months from ruxolitinib initiation to IFI onset in our review (Table 2). Thus, there has been debate about whether the degree of immunosuppression from ruxolitinib mandates any infectious disease screening or the initiation of antimicrobial prophylaxis [40,42,43]. Given the relative infrequency of recognized IFIs, we believe a more proactive approach that includes routine screening and antifungal prophylaxis should be reserved only for those with high-risk exposure or prior history of opportunistic infections, including fungal infections.

Once an IFI is diagnosed, the appropriate course of ruxolitinib therapy remains unclear. Although a few patients resumed or continued ruxolitinib [12,25,28,30], ruxolitinib was discontinued permanently in most cases (76%, Table 2). This approach was also common in patients who had tuberculosis, herpes zoster, or hepatitis B reactivation while on ruxolitinib [15,17]. Currently, there is no large-scale study about switching ruxolitinib to pacritinib (JAK 2-selective inhibitor with virtually no JAK 1 activity) or fedratinib (relatively selective for JAK 2 over JAK 1) in MF patients with IFIs. To our knowledge, our patient is the first who developed disseminated mycosis (histoplasmosis) as a complication of ruxolitinib and has, to date, not experienced a relapse of infection after switching to the more selective JAK2 inhibitor fedratinib. Although clinical data are lacking, the rationale for this switch was based on the theoretical consideration that the immunosuppressive effects of ruxolitinib stem primarily from its inhibition of JAK1 rather than JAK2. While pacritinib is even more selective for JAK2 than fedratinib, with virtually no anti-JAK1 activity, fedratinib was selected in our patient with a preserved platelet count (>100 × 10^9^/L) because of more robust efficacy data for fedratinib in non-cytopenic MF patients [44]. 

Additionally, other strategies, such as adjuvant therapy with granulocyte macrophage colony-stimulating factor, interferon-gamma, or other immunomodulators [45,46], might be helpful in MF patients who need to continue JAK inhibitor therapy. There is a need for thorough studies of the impact of various immunotherapies that boost antifungal immunity in patients with hematological malignancies who have developed IFIs while receiving targeted therapies [47].

In recent years, the epidemiology of IFI in cancer patients has changed significantly, influenced by advances in antineoplastic therapies, improved early detection and identification methods (such as positron emission tomography, matrix-assisted laser desorption ionization-time of flight mass spectroscopy, and whole-genome sequencing), and the introduction of novel antifungal agents for both treatment and prophylaxis [48,49]. New immunotherapeutic and molecularly targeted agents acting on specific molecular pathways are generally better tolerated and more effective than traditional chemotherapy [11]. However, the downstream consequences of inhibiting these pathways may not be fully understood in randomized controlled trials in view of the patient selection and the fact such trials exclude patients with active infections. As a result, significant or unique infectious complications, including IFIs, may only become apparent when these therapies are administered to larger, more diverse populations beyond the confines of clinical trials. This poses substantial challenges in assessing the individual risk associated with each therapeutic agent. Therefore, it is imperative to deepen our understanding of each agent’s specific risks for developing IFIs and the natural history of these infections in patients with hematologic malignancies receiving various targeted therapies. A more standardized approach for capturing IFIs with immunogenetic and immunophenotyping strategies would allow for the accurate assessment of infection risk in individual patients and provide personalized approaches to prevent opportunistic fungal diseases associated with molecularly targeted agents [50].

In the United States, *Histoplasma capsulatum* was previously considered highly endemic to the Ohio and Mississippi River Valleys, a notion established in the 1970s based on antigen skin testing [51]. However, studies in the 2020s have shown that the distribution of histoplasmosis extends beyond these historical boundaries [51]. The expansion of geographic distribution over the past half-century can be attributed to multiple factors, including improvements in diagnostic methods, increased travel, climate change, and changes in land development practices [51,52,53]. Although travel-related fungal infections have often been detected in outbreak settings rather than through routine surveillance, travel-related exposure is considered a significant contributor to the occurrence of dimorphic mycoses [51,54]. In our case, travel to Puerto Vallarta, Mexico, a renowned bat-watching site, is the most likely source of the patient’s *Histoplasma capsulatum* exposure rather than her residence in Texas. Our patient’s isolate belonged to the Latin American group A (LAm A), the predominant clade in Latin America [52]. This contrasts with the United States, where the common clades of Histoplasma capsulatum are North American Classes 1 and 2 (NAm1 and NAm2) [52]. The use of whole-genome sequencing for fungal identification to the clade level will help clinicians better understand species distribution and confirm travel-related fungal infections [55]. *Histoplasma capsulatum* grew in soil enriched with bat guano and may even infect bats [56]. Exposures occurred during activities such as construction, renovation, demolition, soil excavation, spelunking, and cleaning sites harboring the fungus [54,57].

There are several important teaching points in our patient’s diagnosis and management of histoplasmosis. First, the sensitivity of the *Histoplasma* urine antigen tests varies across different assays, and the assay used in our hospital yielded a false-negative result [58]. In addition, our patient also did not elicit *Histoplasma* serum antibodies, which could be attributed to impaired humoral and T-cell immunity resulting from ruxolitinib [59]. Therefore, the borderline positive 1,3-beta-d-glucan was the only positive serum fungal biomarker for an IFI in our case, consistent with reports that *Histoplasma*, as many fungi, can produce 1,3-beta-d-glucan [60]. Second, our patient possibly developed posaconazole-induced pseudohyperaldosteronism [61,62]. However, we did not perform laboratory testing (11-deoxycortisol, plasma aldosterone, and renin activity) for confirmation. Itraconazole and posaconazole are more commonly associated with pseudohyperaldosteronism than fluconazole, voriconazole, and isavuconazole [63]. In our institution, we previously reported a patient who developed posaconazole-induced pseudohyperaldosteronism, in whom the symptoms resolved upon switching to isavuconazole [62]. Third, itraconazole is the recommended step-down therapy for disseminated histoplasmosis following initial improvements with amphotericin B [64]. However, our patient developed worsening symptoms and new mediastinal lymphadenopathy during treatment with itraconazole. Subsequently, the patient exhibited clinical improvement with isavuconazole, administered concurrently with fedratinib. Although isavuconazole appears to be a safe and effective treatment for histoplasmosis [65], the data supporting its superiority over itraconazole in patients receiving JAK inhibitors are lacking. Antifungal susceptibility of *Histoplasma capsulatum* was not tested in this case. Although in vitro antifungal susceptibility testing for dimorphic fungi remains unstandardized. Although epidemiological cut-off values have been proposed, no clinical susceptibility breakpoints have been determined for these organisms [66]. Few in vitro studies have demonstrated low minimum inhibitory concentrations for most mold-active triazoles, including isavuconazole against *Histoplasma capsulatum* [67,68,69]. Fourth, we do not have in-hospital guidelines for treating disseminated histoplasmosis in cancer patients, but we follow the Infectious Diseases Society of America clinical practice guidelines for the management of patients with histoplasmosis with some modifications in cancer patients [64]. We usually start cancer patients with disseminated and serious fungal infections on combination therapy with liposomal amphotericin B and an azole until therapeutic azole levels are reached.

Our review has several limitations. First, only proven IFI cases were analyzed in-depth in this study, which underestimates the incidence of IFIs in patients who received ruxolitinib. However, at the time of this review, no study focused on patients with possible or probable IFIs who received ruxolitinib. Second, ruxolitinib is used to treat MF/PV and GVHD. In this review, we did not discuss stem cell transplant recipients who received ruxolitinib as part of GVHD management because these patients were usually previously exposed to or concomitantly received other immunosuppressants. For instance, we found one case of disseminated aspergillosis in a patient with acute myeloid leukemia who underwent hematopoietic stem cell transplantation and received tacrolimus, ruxolitinib, and budesonide for GVHD [70]. Third, whether ruxolitinib-associated IFIs are time-dependent, dose-dependent, or neither remains to be seen. Well-designed studies or registries are warranted to evaluate the epidemiology and risk of ruxolitinib-associated IFIs. Fourth, IPSS or other MF prognostic scores were unavailable in the reviewed cases, limiting our ability to examine the correlation between IFIs and MF severity. 

In summary, although uncommon, IFIs can complicate ruxolitinib therapy for MF, a hematologic disease historically considered to be associated with very low risk of IFIs in the pre-JAK inhibitor era [39]. Thus, physicians should have a high index of suspicion for potential IFIs in patients on ruxolitinib to facilitate early diagnosis and initiation of appropriate antifungal therapy. Screening and prophylaxis for fungal infections in patients receiving ruxolitinib should be considered based on patient-specific factors. Furthermore, the knowledge gaps identified in our review underscore the critical global need to study the epidemiology, pathobiology, early diagnosis, and multimodal therapy of IFIs in patients with hematological malignancies receiving targeted therapies. 

## Figures and Tables

**Table 1:** Incidence of invasive fungal infection in patients who received ruxolitinib.

Author, Year, Reference	Study Type	Type of Disease	Incidence
Polverelli, 2016 [7,8]	prospective	myelofibrosis	0.6% (3/507) ^a^
Verstivsek, 2017 [9]	clinical trial	myelofibrosis	2.6% (4/155) ^b^
Kusne, 2020 [10]	retrospective	Not available ^c^	3.7% (5/135) ^d^
Gold, 2022 [11]	retrospective	Not available ^c^	4.9% (26/531) ^e^

a. Candidiasis (1); aspergillosis (2). b. Details of fungal infections are not available. c. In these studies, ruxolitinib was used for polycythemia vera, essential thrombocythemia, myelofibrosis, and graft-vs-host disease. d. Coccidioidomycosis (5). This study specifically examined the incidence of coccidioidomycosis in patients who received ruxolitinib. e. Candidiasis (6); aspergillosis (5); *Pneumocystis jiroveci* pneumonia (3); coccidioidomycosis (1); cryptococcosis (1); histoplasmosis (1); not specified (9).

**Table 2:** Clinical presentation of 28 myelofibrosis patients on ruxolitinib with invasive fungal infection.

Patient Characteristic	
**Sex**	
Male	18 (69%) ^a^
**Age, median year (IQR)**	70 (65–73) ^a^
**MF type**	
Primary MF	15 (54%)
Post-PV MF	5 (18%)
Post-ET NF	2 (7%)
Unknown	6 (21%)
**Associated conditions**	
Splenomegaly at the onset of IFI	11 (39%)
Diabetes mellitus	2 (7%)
**Co-infection**	5 (18%) ^b^
**Time from initiation of ruxolitinib to the onset of IFI, medium months (IQR)**	11 (6–27) ^c^
**Ruxolitinib treatment after diagnosis of IFI ^d^**	
Discontinued permanently	13 (76%)
Discontinued and restarted once IFI resolved	3 (18%)
Continued	1 (6%)
**Antifungal prophylaxis at the onset of IFI**	0 (0%)
**IFI, location**	
Disseminated	11 (39%)
Pulmonary	6 (21%)
Central nervous system	5 (18%)
Other localized infection ^e^	6 (21%)
**Fungal infection, pathogen**	
*Cryptococcus* spp.	13 (46%)
*Coccidioides* spp.	3 (11%)
*Pneumocystis jirovecii*	3 (11%)
Others ^f^	9 (32%)
**All-cause mortality**	5 (24%) ^g,h^

a. Data are available for 26 patients. b. One case each of *Klebsiella pneumoniae* bacteremia, pulmonary tuberculosis, pneumonia due to *Mycobacterium avium* complex, and *Mycobacterium haemophilum* skin infection. One patient has disseminated tuberculosis and shingles (herpes zoster). c. Data are available for 22 patients. d. Data are available for 17 patients. e. One case each of intestinal candidiasis, rhino-orbital mucormycosis, skin abscess due to *Pleurostoma richardsiae*, cutaneous coccidioidomycosis, cutaneous sporotrichosis, and cutaneous phaeohyphomycosis (Exophiala sp.). f. One case each is due to *Aspergillus* sp., *Candida albicans*, *Exophiala* sp., *Histoplasma capsulatum*, Mucorales, *Pleurostoma richardsiae*, *Sporothrix schenckii*, *Talaromyces marneffei*, and *Verruconis gallopava*. g. Data are available for 21 patients. h. 5 patients expired on days 7, 14, 22, 48, and 65 of hospitalization.

## Data Availability

The de-identified datasets generated during and/or analyzed during the current study are available from the corresponding author upon reasonable request. The data are not publicly available due to ethical reasons.

## Supplementary Materials

The following supporting information can be downloaded at: https://www.mdpi.com/article/10.3390/jof10040264/s1.
