# Supplementary material for: Disseminated Histoplasmosis in a Patient with Myelofibrosis on Ruxolitinib: A Case Report and Review of the Literature on Ruxolitinib-Associated Invasive Fungal Infections
Source: J Fungi (Basel). 2024 Mar 31;10(4):264. doi: 10.3390/jof10040264 (PMC11051496; doi:10.3390/jof10040264)
Supplement: Supplementary file 1 [file jof-10-00264-s001.zip › jof-2909918-supplementary.pdf]

Ovid-Embase:

1. exp Histoplasmosis/ or exp histoplasma/ or exp fungi/ or exp aspergillus/ or trichosporon/
2. (aspergil* or trichospor*).ti,ab,kf.
3. Histoplasm*.ab,kf,ti.
4. exp Blastomyces/ or exp Blastomycosis/
5. blastomyc*.ab,kf,ti.
6. exp Coccidioides/ or exp Coccidioidomycosis/
7. Coccidioid*.ab,kf,ti.
8. exp Meningitis, Cryptococcal/ or exp Cryptococcosis/ or exp Cryptococcus/ or exp Cryptococcus neoformans/ or exp Cryptococcus gattii/
9. Cryptococc*.ab,kf,ti.
10. exp Talaromyces/
11. Talaromyc*.ab,kf,ti.
12. Penicilliosis.ab,kf,ti.
13. exp Paracoccidioides/ or exp Paracoccidioidomycosis/
14. Paracoccidioid*.ab,kf,ti.
15. exp Sporotrichosis/ or exp Sporothrix/
16. sporot*.ab,kf,ti.
17. exp mucor racemosus/ or exp mucorales/ or exp zygomycetes/ or exp absidia/ or exp amylomyces/ or exp apophysomyces/ or exp blakeslea/ or exp cunninghamella/ or exp lichtheimia/ or exp mortierella/ or exp mucor/ or exp mycotypha/ or exp phycomyces/ or exp rhizomucor/ or exp rhizopus/ or exp syncephalastrum/ or exp umbelopsis/ or exp mucormycosis/
18. (mucor* or Rhizopus or zygomycet* or absidia or amylomyces or apophysomyc* or blakeslea or cunninghamella or lichtheimia or mortierella or mycotypha or phycomyces or rhizomucor* or syncephalastrum or umbelopsis).af.
19. or/1-18 [fungal infections]
20. exp ruxolitinib/
21. (ruxolitinib or jakafi).af.
22. 20 or 21
23. 19 and 22
24. myelofibrosis.af.
25. 23 and 24

Ovid-Medline:

1. exp Histoplasmosis/ or exp histoplasma/ or exp fungi/ or exp aspergillus/ or trichosporon/
2. (aspergil* or trichospor*).ti,ab,kf.
3. Histoplasm*.ab,kf,ti.
4. exp Blastomyces/ or exp Blastomycosis/
5. blastomyc*.ab,kf,ti.
6. exp Coccidioides/ or exp Coccidioidomycosis/
7. Coccidioid*.ab,kf,ti.
8. exp Meningitis, Cryptococcal/ or exp Cryptococcosis/ or exp Cryptococcus/ or exp Cryptococcus neoformans/ or exp Cryptococcus gattii/
9. Cryptococc*.ab,kf,ti.
10. exp Talaromyces/
11. Talaromyc*.ab,kf,ti.
12. Penicilliosis.ab,kf,ti.
13. exp Paracoccidioides/ or exp Paracoccidioidomycosis/
14. Paracoccidioid*.ab,kf,ti.
15. exp Sporotrichosis/ or exp Sporothrix/
16. sporot*.ab,kf,ti.
17. exp mucor racemosus/ or exp mucorales/ or exp zygomycetes/ or exp absidia/ or exp amylomyces/ or exp apophysomyces/ or exp blakeslea/ or exp cunninghamella/ or exp lichtheimia/ or exp mortierella/ or exp mucor/ or exp mycotypha/ or exp phycomyces/ or exp rhizomucor/ or exp rhizopus/ or exp syncephalastrum/ or exp umbelopsis/ or exp mucormycosis/
18. (mucor* or Rhizopus or zygomycet* or absidia or amylomyces or apophysomyc* or blakeslea or cunninghamella or lichtheimia or mortierella or mycotypha or phycomyces or rhizomucor* or syncephalastrum or umbelopsis).af.
19. or/1-18 [fungal infections]
20. exp Janus Kinase Inhibitors/
21. (ruxolitinib or jakafi).af.
22. 20 or 21
23. 19 and 22
24. myelofibrosis.af.
25. 23 and 24

Pubmed:

("invasive fungal infections"[MeSH Terms] OR "mycoses"[MeSH Terms] OR "histoplasmosis"[MeSH Terms] OR "histoplasma"[MeSH Terms] OR "aspergillus"[MeSH Terms] OR "blastomyces"[MeSH Terms] OR "blastomycosis"[MeSH Terms] OR "coccidioides"[MeSH Terms] OR "coccidioidomycosis"[MeSH Terms] OR "cryptococcosis"[MeSH Terms] OR "cryptococcus"[MeSH Terms] OR "talaromyces"[MeSH Terms] OR "paracoccidioides"[MeSH Terms] OR "paracoccidioidomycosis"[MeSH Terms] OR "mucorales"[MeSH Terms] OR "mucormycosis"[MeSH Terms] OR ("fungus"[Text Word] OR "fungi"[Text Word] OR "fungal"[Text Word] OR "histoplasm*"[Text Word] OR "aspergil*"[Text Word] OR "trichosporon"[Text Word] OR "blastomyc*"[Text Word] OR "coccidioid*"[Text Word] OR "cryptococ*"[Text Word] OR "talaromyc*"[Text Word] OR "mucor*"[Text Word] OR "cunninghamella"[Text Word] OR "rhizomucor*"[Text Word])) AND ("janus kinase inhibitors"[MeSH Terms] OR ("ruxolitinib"[Text Word] OR "jakafi"[Text Word] OR "janus kinase inhibitor*"[Text Word])) AND ("primary myelofibrosis"[MeSH Terms] OR "myelofibrosis"[Text Word])

Scopus:

TITLE-ABS-KEY (("invasive fungal infections" OR "mycoses" OR "histoplasmosis" OR "histoplasma" OR "aspergillus" OR "blastomyces" OR "blastomycosis" OR "coccidioides" OR "coccidioidomycosis" OR "cryptococcosis" OR "cryptococcus" OR "talaromyces" OR "paracoccidioides" OR "paracoccidioidomycosis" OR "mucorales" OR "mucormycosis" OR ("fungus" OR "fungi" OR "fungal" OR "histoplasm*" OR "aspergil*" OR "trichosporon" OR "blastomyc*" OR "coccidioid*" OR "cryptococ*" OR "talaromyc*" OR "mucor*" OR "cunninghamella" OR "rhizomucor*")) AND ("janus kinase inhibitors" OR ("ruxolitinib" OR "jakafi" OR "janus kinase inhibitor*")) AND ("myelofibrosis"))
